# Advances in biotechnology of *Emblica officinalis* Gaertn. syn. *Phyllanthus emblica* L.: a nutraceuticals-rich fruit tree with multifaceted ethnomedicinal uses

**DOI:** 10.1007/s13205-020-02615-5

**Published:** 2021-01-11

**Authors:** Saikat Gantait, Manisha Mahanta, Soumen Bera, Sandeep Kumar Verma

**Affiliations:** 1 Crop Research Unit (Genetics and Plant Breeding), Bidhan Chandra Krishi Viswavidyalaya, Mohanpur, Nadia, 741252 West Bengal India; 2 College of Agriculture, Bidhan Chandra Krishi Viswavidyalaya, Burdwan, 713101 West Bengal India; 3 Institute of Biological Science, SAGE University, Baypass Road, Kailod Kartal, Indore, 452020 Madhya Pradesh India

**Keywords:** Amla, Callus, Emblicanin, Micropropagation, Molecular markers, Nanoparticles, Somatic embryogenesis

## Abstract

*Emblica officinalis* Gaertn. syn. *Phyllanthus emblica* L., universally known as ‘Amla’ or ‘Aonla’ or ‘Indian gooseberry’, is a popular fruit tree belonging to the family Euphorbiaceae and order Geraniales. It is said to be the very first tree that originated on earth, as claimed by age-old Indian mythology. Almost all parts of the tree *i.e.,* root, bark, leaf, flower, fruit and seed are utilized in Ayurvedic and Unani medicinal formulations to improve the overall digestive process, decrease fever, act as a blood purifier, relieve asthma and cough, improve heart health, etc. This tree contains major secondary metabolites like emblicanin-A and emblicanin-B, and also is an affluent source of vitamin-C. Additionally, some other secondary metabolites like tannins, gallic acid, pyrogallol, and pectin are also present in significant amounts. Conventional propagation has been improved via suitable interventions of agrotechnology both in production and protection areas. However, the rate of propagation remains slower; therefore, attempts have been made for biotechnological advancements on *E. officinalis*. The present review makes an attempt to highlight the botanical description, geographical distribution, ethnopharmacological importance, conventional propagation and protection of this medicinal tree, describing the in vitro-based plant organ and tissue culture methods like direct and indirect organogenesis and somatic embryogenesis along with interventions of molecular marker-based biotechnology and nanotechnology. Further, the prospect of the yet-to-be-explored biotechnological methods for secondary metabolite enhancement like cell suspension, protoplast culture, genetic transformation, etc. and their potential for enhanced emblicanin production have also been discussed in this appraisal.

## Introduction

*Emblica officinalis* Gaertn. (synonym *Phyllanthus emblica* L.) (Euphorbiaceae family) is a deciduous tree, popular as ‘Amla’ or ‘Aonla’ or ‘Indian gooseberry’. ‘Amla’ tree is said to be the very first tree that originated on earth, as claimed by age-old Indian mythology. The fruit of this tree is a reservoir of various nutraceuticals like calcium, vitamin-C, lysine, minerals, methionine, nicotinic acid, phosphorus, riboflavin, tryptophane and is said to have immune-boosting efficiency against multiple diseases and are also extensively applied in *Ayurveda*, an Indian ancient system of medicine (Bhagat 2014). Even if the fruit is eaten in its unripe state, it is considered to be beneficial for health*.* It has also found its application in the food processing, pharmaceutical, and cosmetic sectors*. E. officinalis* tree is well suited to grow even in saline–sodic and other wasteland soil conditions; and the fruits remain in season for almost ten months*.* Nowadays, *E. officinalis* is one of the preferred species for small-scale or marginal-farm-based agro-forest industries in multiple tropical and sub-tropical countries, owing to its high nutraceutical factors and its versatility to be processed into a wide range of pharmaceutical products (Pathak et al. 2003).

The natural propagation frequency of *E. officinalis* is quite low and the trees are highly prone to several pests and pathogens. Quite a few attempts have been made towards the biotechnological improvement of *E. officinalis* for the past two and half decades, with special emphasis on its mass propagation under in vitro conditions, eventually to yield pathogen-free quality planting materials. However, the reports on the biotechnological improvement of this tree are insufficient to date and a number of strategies are yet to be explored to enrich this highly valued medicinal tree by enhancing the production of its nutraceuticals. In such a backdrop, the present review aims to highlight the significance of applications of *E. officinalis* in pharmaceutical industries and multiple in vitro biotechnological strategies that were adopted for its genetic improvement and mass propagation, for instance, organogenesis (both direct and indirect), somatic embryogenesis, etc. Likewise, the possibilities and prospects of an array of unexplored in vitro biotechnological tools and techniques (such as cell suspension, elicitation, synthetic seed production, hairy root culture, cryopreservation, etc.) have been discussed in a precise way to help the readers in designing their future experiments on the biotechnological improvement of this nutraceutical-rich tree.

### Geographical distribution

**Fig. 1:**
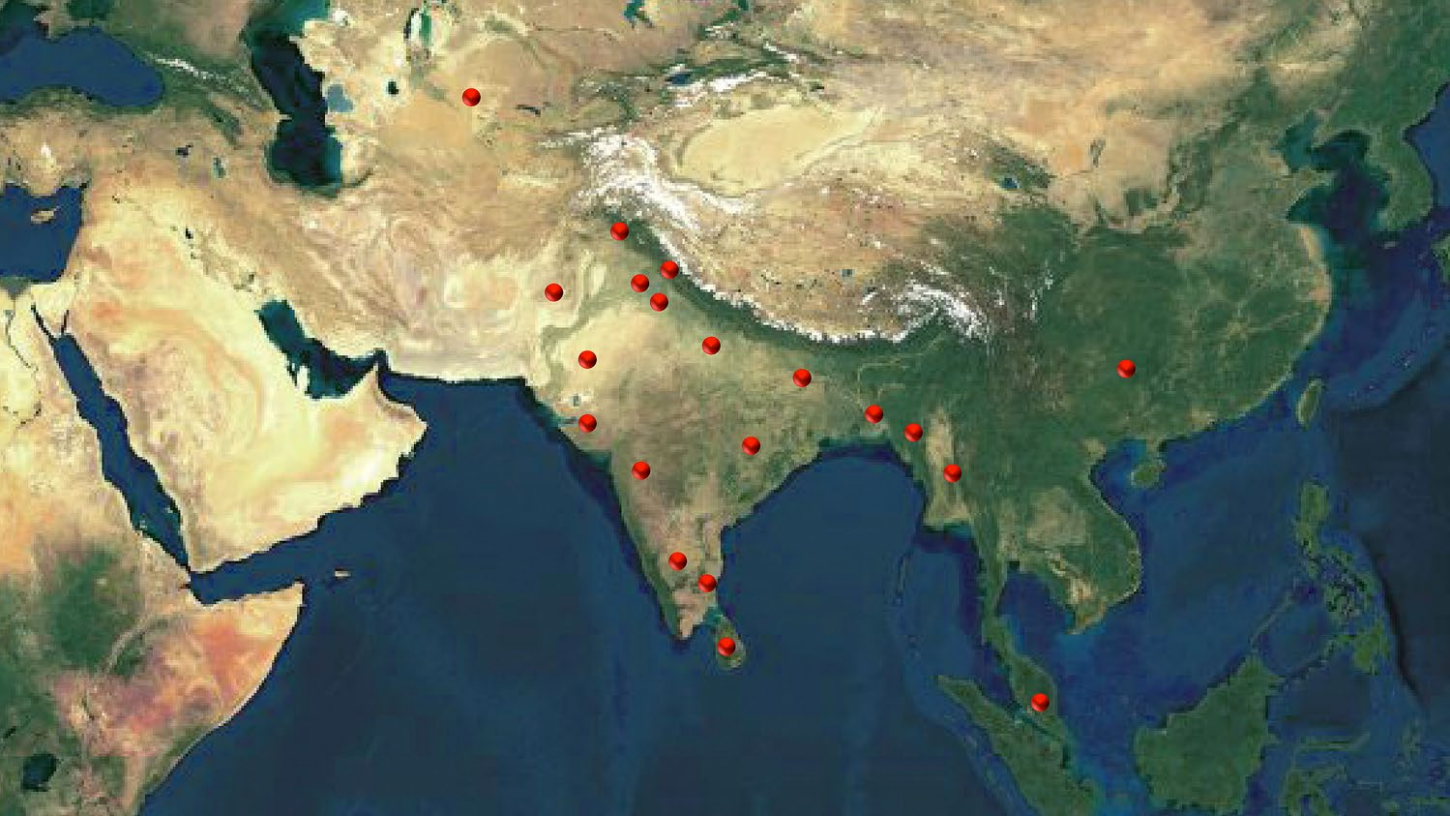
Global distribution of *Emblica officinalis* Gaertn. syn. *Phyllanthus emblica* L. (Photograph is not in scale) (*Source*: unpublished photograph of Saikat Gantait)

*E. officinalis*, originating from India, is also cultivated in several other tropical and sub-tropical countries (Fig. 1) such as Bangladesh, China (southern part), Malaysia, Mascarene Islands, Myanmar, Pakistan, Sri Lanka, and Uzbekistan (Thilaga et al. 2013). In India, this tree can usually be found in the coastal, tropical, sub-tropical districts and on hill slopes up to a height of 200 m and up to 4500 ft in the hills. It is also cultivated in the plain land and hilly areas of the valley of Kashmir (Rai et al. 2012; Thilaga et al. 2013). It prevails abundantly in deciduous forests of India (Sai et al. 2002) but lately, accelerated cultivation of this tree was observed in the semi-arid region as well as in the eastern–south-eastern states of India (Nayak et al. 2010).

### Botanical features

**Fig. 2:**
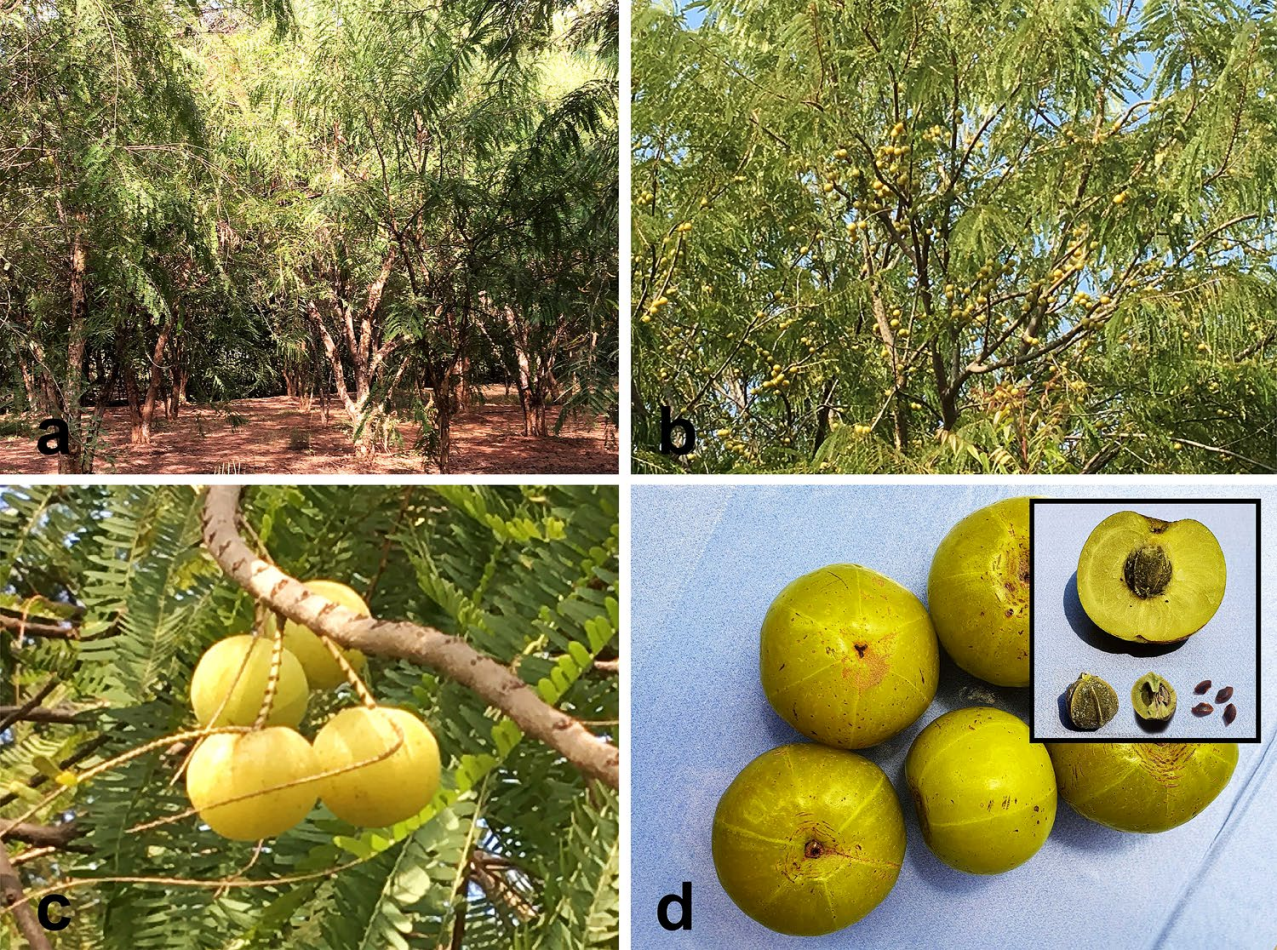
Salient botanical features of *Emblica officinalis* Gaertn. syn. *Phyllanthus emblica* L. **a** Full-grown trees in deciduous forest, **b** fruit-bearing trees with light grayish or greenish-brown barks, **c** arrangement of leaves and fruits along the branchlets of the tree, **d** almost sphere-shaped fruits showing minor conic indentation on both poles and six blurred perpendicular pole-to-pole stripes [Inset: top—edible fleshy yellowish-green mesocarp; left—endocarp in the form of hard stone encasing the seeds; center—crustaceous cocci, right—smooth, dark brown, and trigonous seeds] (Photographs are not in scale) (*Source*: unpublished photographs of Saikat Gantait)

An average *E. officinalis* tree, usually deciduous in nature, is of medium height (8–18 m) (Fig. 2a). The bark is thick (~ 12 mm) with light grayish or greenish-brown in color, highlighting a mottled appearance at maturity (Fig. 2b). The leaves of this tree are pinnate in type, simple, alternate, bifarious, sub-sessile, light green in color and arranged in a close pattern along the branchlets (Fig. 2c). Petioles are striated. February–May mark the flowering period (Rai et al. 2012). The flowers appear in greenish-yellow hue along the axillary fascicles with six-parted calyx (Meena et al. 2010). Male flowers are found abundantly in the axils of lower leaflets; whereas, the female flowers (with the three-celled ovary, three-stigmatic, solitary, sessile nature) are fewer in number and usually found in the most exterior floriferous axils along with some male flowers (Treadway 1994). The *E. officinalis* fruits (measuring 15–20 mm × 18–25 mm) are drupe in nature and almost sphere-shaped with a minor conic indentation on both poles (Fig. 2d). The edible smooth fleshy mesocarp is a pale yellow to yellowish-green in appearance and the endocarp that forms the hard stone encasing the seeds turns yellowish-brown during maturity (Khan 2009; Patel and Goyal 2011). Six blurred perpendicular pole-to-pole lines surrounding six trigonous seeds are observed in two-seeded three-crustaceous cocci (Fig. 2d). Usually, the trees developed from seedlings initiate fruiting around eight years after planting, which is almost three years later than that of the trees developed from budded clones (Kumar et al. 2012a; Rai et al. 2012). The berries generally start to ripen during autumn and each weighs ~ 60–70 g. The fruit is fibrous and tastes almost astringent, bitter and sour (Kumar et al. 2012b). Seeds are smooth, dark brown and found four–six in number; two seeds enclosed in each cell (Fig. 2d).

### Nutraceutical compositions

**Fig. 3:**
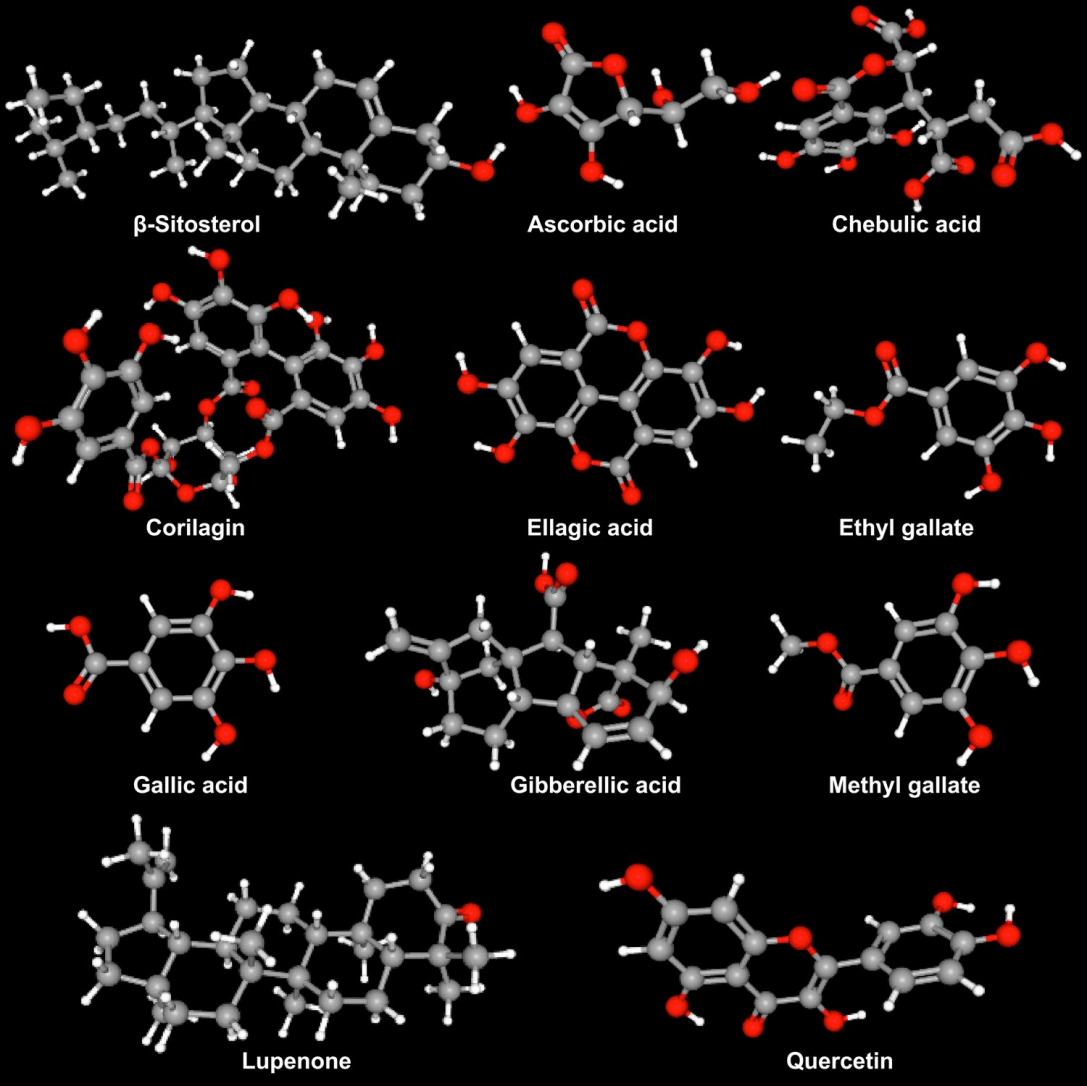
Some of the key phytochemicals found in different parts of *Emblica officinalis* Gaertn. syn. *Phyllanthus emblica* L. (*Structure * *source*: PubChem https://pubchem.ncbi.nlm.nih.gov) (*Source*: unpublished photograph of Saikat Gantait)

Almost all the vegetative and reproductive parts of *E. officinalis* are rich with multiple nutraceuticals (Table 1; Fig. 3), significant of which are iron, calcium, carotene, niacin, phosphorous, riboflavin, thiamine among several others. The seed contains a certain type of fixed oil (yielding ~ 16%) (yellowish-brown in color) comprising several fatty acids viz. 44% linoleic, 28.4% oleic, 8.8% linolenic, 3% palmitic, 2.15% stearic, 1% myristic acid; along with multiple essential oils and phosphatides. The bark of this tree is rich in both tannin and leucodelphinidin; the root is enriched with lupeol and ellagic acid, and a significant amount of tannin is found in the leaf and fruit as well (Bhattacharya et al. 1999). A specific amount of d-fructose, d-glucose, d-myo-inositol and free sugars are found in an ethanol-soluble fraction derived from this fruit; whereas, an acidic water-soluble fraction of the same comprises pectin with residues of d-arabinosyl, d-galacturonic acid, d-galactosyl, d-glucosyl, d-mannosyl, d-rhamnosyl, and d-xylosyl (Bhattacharya et al. 1999). Emblicanin-A and Emblicanin-B (two low molecular weight hydrolyzable tannins) accompanied by pedunculagin and punigluconin are the integral components found in *E. officinalis* fruits (Kim et al. 2005; Chaudhary et al. 2020); whereas, tannins, gallic acid and pyrogallol are the active principles of this fruit (Veena and Shanthi 2006).

**Table 1:** Source of different phytochemicals from *Emblica officinalis* Gaertn. syn. *Phyllanthus emblica* L

Plant part	Phytochemical	References
Bark	β-sitosterol, Leucodelphinidin, Lupeol, Tannin	Srikumar et al. (2007), Khan (2009)
Fruit	3–6-di-*O*-galloyl-glucose, Alanine, Ascorbic acid, Aspartic acid, Arginine, β-carotene, Boron, Calcium, Carbohydrates, Chebulagic acid, Chibulinic acid, Chebulaginic acid, Chebulic acid, Chloride, Copper, Corilagic acid, Corilagin, Cystine, d-fructose, d-glucose, Ellagic acid, Emblicanin-A, -B, Emblicol, Ethyl gallate (syn. Phyllemblin), Gallic acid, Gallic acid ethyl ester, Gibberellin A_1_, Gibberellin A_3_ (syn. Gibberellic acid), Gibberellin A_4_, Gibberellin A_7_, Gibberellin A_9_, Glucogallin, Glucose, Glutamic acid, Glycine, Histidine, Iron, Isoleucine, Leucine, Lysine, l-malic acid 2-*O*-gallate, Manganese, Magnesium, Methionine, Myo-inositol, Myristic acid, Niacin, Nitrogen, Pectin, Phenylalanine, Phosphorus, Phyllemblinic acid, Phyllemblic acid, Polysaccharide, Potassium, Proline, Protein, Quercetin, Riboflavin, Rutin, Selenium, Serine, Silica, Sodium, Starch, Sucrose, Sulfur, Tannin, Terchebin, Thiamin, Threonine, Trigalloyl glucose, Tryptophan, Tyrosine Zinc, Zeatin, Zeatin riboside, Zeatin nucleotide, Phyllantidine, Phyllantine	Ghosal et al. (1996), Jagetia et al. (2004), Srikumar et al. (2007), Singh et al. (2011), Srinivasan et al. (2018)
Leaf	Amlaic acid, Astragalin, Ellagic acid, Gallo-tannin, Kaempferol, Kaempferol-3-*O*-glucoside, Phyllantidine, Phyllantine, Rutin, Tannin	Srikumar et al. (2007), Khan (2009)
Root	Ellagic acid, Lupeol	
Seed	β-sitosterol, Flavonoid, Linoleic acid, Linolenic acid, Myristic acid, Oleic acid, Palmitic acid, Stearic acid, Tannin	Srikumar et al. (2007), Khan (2009), Sriwatcharakul (2020)
Shoot	3–6-di-*O*-galloyl-glucose, β-sitosterol, Chebulagic acid, Chibulinic acid, Ellagic acid, Gallic acid, Glucogallin, Lupeol	Srikumar et al. (2007)
Twig	Tannin	
Whole plant	Ascorbic acid, Lupenone

### Pharmaceutical uses

**Fig. 4:**
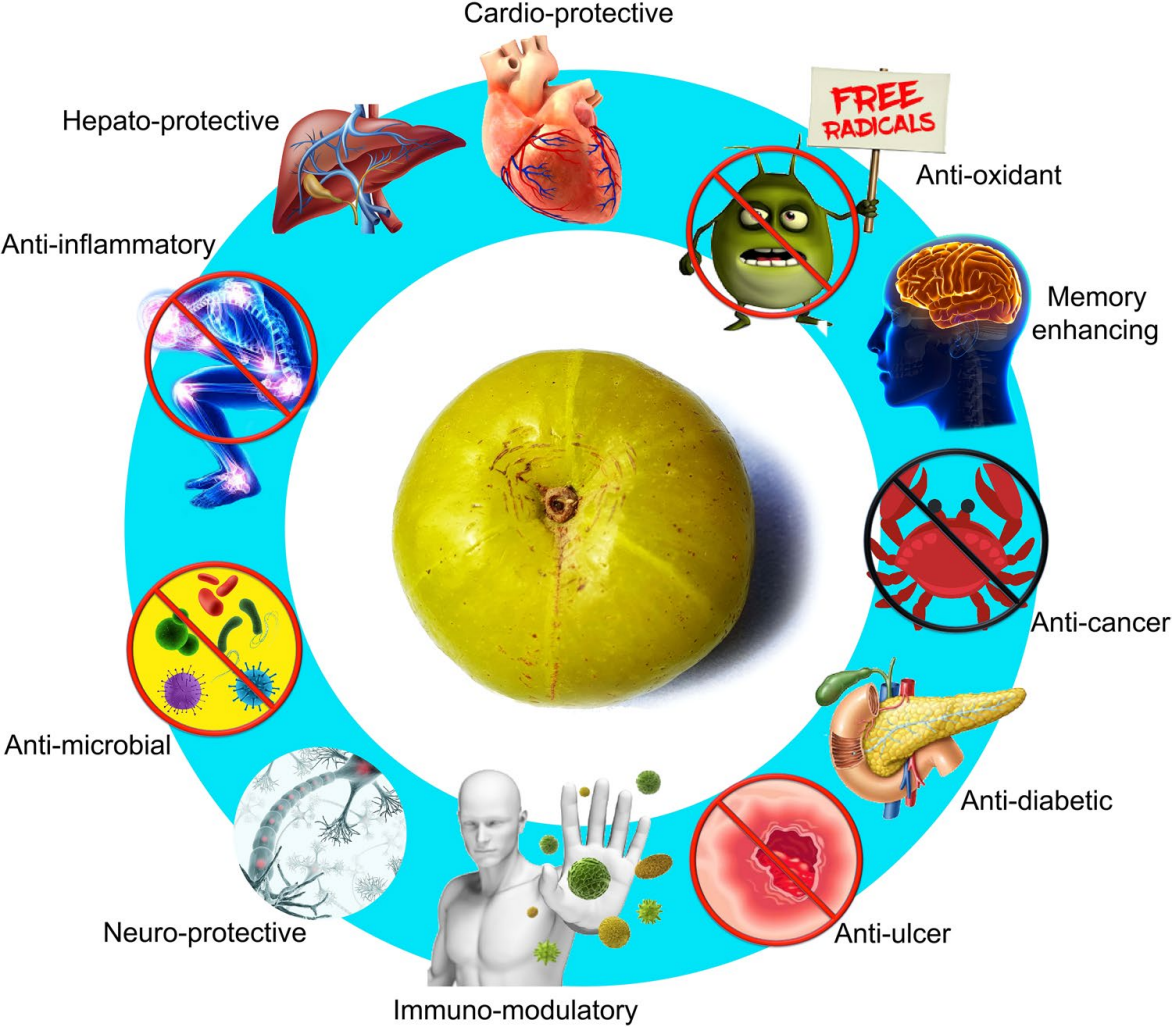
Diagrammatic presentation of selected pharmacological properties of *Emblica officinalis* Gaertn. syn. *Phyllanthus emblica* L. (*Source*: unpublished photograph of Saikat Gantait)

Extracts and various herbal preparations of *E. officinalis* exhibited their disease-fighting potential against an array of health issues in a comparable way to that of the usual medications. It is because of its multifaceted ethnic, ethnopharmaceutical, and ethnobotanical importance, all the parts of the tree, including the bark, flower, fruit, seed, root, either fresh or dried, are used in the Indian traditional system of Ayurvedic or Unani medicine (Khan 2009; Kumar et al. 2012b). Concise information on the multifaceted pharmacological properties has been presented (Table 2; Fig. 4).

**Table 2:** Some major pharmacological properties of *Emblica officinalis* Gaertn. syn. *Phyllanthus emblica* L

Property	References
Anti-aging	Pal et al. (2017)
Anti-cancerous	Mahata et al. (2013), Wiart (2013)
Anti-diabetic	Kumar et al. (2012b), Nain et al. (2012), Kalekar et al. (2013), Fatima et al. (2017), Srinivasan et al. (2018); Sharma et al. (2020)
Anti-microbial	Saeed and Tariq (2007), Srikumar et al. (2007), Nath et al. (2012), Dinesh et al. (2017), Singh et al. (2019a)
Anti-mutagenic	Sumitra et al. (2009), Agrawal et al. (2012)
Anti-pyretic, analgesic, anti-inflammatory	Mythilypriya et al. (2007), Muthuraman et al. (2011), Gupta et al. (2013), Asmilia et al. (2020)
Anti-oxidant	Bafna and Balaraman (2005), Dhanalakshmi et al. (2007); Golechha et al. (2012), Nain et al. (2012), Rose et al. (2018), Singh et al. (2019b), Majeed et al. (2020)
Anti-ulcerous, wound healing	Sai et al. (2002), Bafna and Balaraman (2005), Mehrotra et al. (2011), Chatterjee et al. (2012), Chularojmontri et al. (2013)
Cardio-protective	Ojha et al. (2012), Rajak et al. (2004)
Chemo-protective	Sharma and Sharma (2011)
Hepato-protective	Sultana et al. (2005), Pramyothin et al. (2006), Vasant and Narasimhacharya (2012), Sarkar et al. (2015), Baliga et al. (2019)
Immuno-modulatory	Nemmani et al. (2002), Sai et al. (2002), Srikumar et al. 2005, Patel et al. (2017)
Memory enhancing	Vasudevan and Parle (2007), Ali et al. (2013)
Neuro-protective	Ashwlayan and Singh (2011), Reddy et al. (2011), Xie et al. (2012), Mathew and Subramanian (2014), Shalini and Sharma (2015), Justin Thenmozhi et al. (2016)

The fruit may also be utilized in extending longevity while acting as a rejuvenating agent, and conventionally improves the overall digestive process by managing constipation as well. It further decreases fever, acts as a blood purifier, relieves asthma and cough, improves heart health, and has invigorating effects on hair growth. *E. officinalis* fruits are one of the wealthiest natural reservoirs of vitamin-C that plays a crucial part in counteracting an ample number of ailments (Bhagat 2014). They are beneficial in treating dyslipidemia (Upadya et al. 2019), cancer, chronic periodontitis, dental caries, hyperacidity, hypertension, inflammation, iron deficiency anemia, neurological disorders, obesity, osteoporosis, pulmonary TB, skin diseases, Type 2 diabetes, Type II hyperlipidemia, vitiligo in addition to lifestyle diseases, parasitic and other infectious disorders (Variya et al. 2016; Yadav et al. 2017).

It is noteworthy to mention that *E. officinalis* is a usual component in Ayurvedic preparations (multi-herbal), and especially it is the key constituent in an ancient herbal formulation known as “*Chyawanprash*”, a premier rejuvenating compound that was first cited in the “*Charaka Samhita*”. This formulation is comprised of as many as 43 herbal ingredients along with sugarcane juice, sesame oil, honey, and clarified butter. The other vital and commonly used Ayurvedic herbal mixture is “*Triphala* (means three fruits) *Churna* (means dust)” that contains equivalent shares of *Terminalia bellirica*, *T. chebula* and *P. emblica* (devoid of seeds). “*Triphala Churna*” is taken as a light purgative that purifies the gastro-intestinal tract of the human body (Jagetia et al. 2004).

### Conventional propagation

#### Climate and soil

*E. officinalis* is a hardy sub-tropical fruit-bearing tree and is propagated throughout a wide range of climatic conditions. However, large-scale cultivation of this tree in tropical and dry sub-tropical climate is rather effective (Sengupta et al. 2020). For optimum growth and development of *E. officinalis* tree, 630–800 mm annual rainfall is most favorable, and it can thrive up to 46 °C since the warm climate is quite advantageous during the onset of its fruit growth (Wali et al. 2015). Being a deciduous and deep-rooted tree species, it has the plasticity to be grown in a broad range of soil type (from sandy loam to clay) and in arid to semi-arid regions. The immense potential of *E. officinalis* has also been proven for commercial-scale cultivation in the salt-affected region, since as large as ~ 7 million ha of saline wasteland (Wali et al. 2015) as well as an extent of ravine land (Das et al. 2011) in India is covered by this tree. Growers can gain marked profit via its cultivation even in marginal lands wherein this hardy tree grows well too (Sengupta et al. 2020).

#### Important cultivars

In various institutes in India, some promising cultivars of *E. officinalis* have been developed, for instance, ‘NA-10’, ‘NA-9’, ‘NA-8’, ‘NA-7’, ‘NA-6’, ‘Laxmi-52’, ‘Krishna’, ‘Kanchan’, ‘Goma Aishwarya’, ‘Gujarat-2’, ‘Gujarat-1’, ‘Francis’, ‘Chakaiya’, ‘BSR-1’, and ‘Banarasi’ (More et al. 2008); and among these cultivars, ‘Kanchan’ is the highest yielder that produces the best quality fruit (Maholiya et al. 2015).

#### Seed propagation

*E. officinalis* trees that are usually raised from seeds produce inferior quality fruit and exhibit long juvenile growth stage. The trees raised by self-sown seeds in forests are not true-to-type and exhibit high variability in terms of irregular pattern of vegetative growth, fruit shape, size, yield (both quality and quantity), etc.; besides, such trees are late-bearing since these need extended time to attain first reproductive stage than that of the vegetatively propagated ones (Sampath Kumar et al. 2012). Moreover, owing to the hard and thick testa, fresh seeds usually do not germinate even if exposed to favorable conditions and consequently, require specific treatments like water-soaking, scarification, stratification, plant growth regulator-treatment, etc. to overcome dormancy (Barathkumar 2019). In regular seed-propagation practice, mature fruits from seed-borne plants are plucked mainly in the months of November–December. Then, the collected fruits are usually sun-dried, so that seeds can be pulled out with gentle pressure. One quintal of indigenous fruits typically yields 1 kg seeds. Standard test weight (of 1000 seeds) of *E. officinalis* fresh seeds ranges from 20 to 33 g (Wali et al. 2015). The conventional time of sowing of *E. officinalis* seeds is between April and June. The seeds are usually sown in small polybags at the depth of 5 cm (Singhal et al. 2017). It is evident that immersion of seeds in GA_3_ 200 ppm solution for 12 h, subsequently 12-h shade drying significantly improved seed germination, seedling health and vigor. The enzymatic and hormonal mechanism stimulates multiple metabolic activities that result in elongation of shoot and root, as well as increased dry weight of seedlings (Chiranjeevi et al. 2017).

#### Budding and grafting

In the case of *E. officinalis*, budding is the most practical method among all the other modes of vegetative propagation. Amid different budding techniques, patch budding and shield budding are practised for commercial propagation. Generally, one-year-old seedlings having 1 cm thickness are shield budded with healthy and plump buds, during early July. The success rate of shield budding is quite promising than patch budding (Wali et al. 2015). Apart from budding, particularly for dry areas, softwood grafting is successfully followed with a 70% success rate. In addition, cleft and veneer grafting are proven to be effective (Wali et al. 2015; Jalal et al. 2019).

#### Nursery preparation

Seedbed preparation is a pre-requisite for raising seedlings. Generally, nursery beds are 10–15 cm elevated using farmyard manure (FYM), under partial light. During spring or rainy season, pre-soaked (in water for 48 h) seeds are sown at 2–3 cm depth and 2–3 seeds per hill, keeping a spacing of 15 cm (row–row). Germinated healthy plants become set for ultimate planting are utilized as rootstock for budding as well. For rootstock preparation, 6–12-month-old seedlings are usually considered. Ripen fruits are harvested in the months of November–December. However, following April, the extracted seeds are sown in an elevated nursery bed. For subsequent budding, the seedlings are transplanted in individual beds (Wali et al. 2015).

#### Orchard establishment and management

An *E. officinalis* tree initiates to bear fruits after 3–4 years of planting, and following 10–12 years, attains certain level wherein they are seasoned enough for commercial fruit production even up to 60–70 years (if managed adequately). The layout is followed on bush-free, deeply ploughed and leveled land, supplemented with organic matter and green manure crops (like *Sesbania aculeata* or *Crotalaria juncea*). Under sub-tropical climate, planting is mainly started from mid-August and completed within the end of the month itself. Early planting ensures that the plants get a long rainy period, which is necessary for the saplings for their preliminary growth and development. However, the seedlings that are transplanted around mid-July are reported to have the maximum success in budding both during March and July (Banyal and Banyal 2019). Being a deciduous tree, *E. officinalis* attains the height of ~ 8–18 m at maturity. Hence, square planting by maintaining 8–10 m gap within and between the rows is followed to facilitate sufficient light penetration, smooth cultural operations (like pruning), and adequate fruiting. Hedgerow planting is also being considered nowadays, wherein 8 m line-to-line and 4–5 m plant-to-plant gap is maintained. *E. officinalis* is typically propagated directly via seedlings under adverse soil conditions in suitable containers, followed by transplantation of the same at a permanent site, and subsequently in situ budding is carried out. It is necessary for two cultivars to be planted in alternate rows to overcome self-incompatibility. The fields are laid out and marked before planting. Then, pits of 1 × 1 × 1 m^3^ size are excavated at indicated places in the month of April–May and left exposed for at least two weeks (for insect pests eradication). Pits are then loaded with FYM (~ 15–20 kg), neem cake (1 kg), muriate of potash (MoP) (200–300 g), single superphosphate (500 g) along with Furadan 3G® and Heptachlor®, before the rainy season arrives. Thereafter, with the first few showers of rain, the soil is left as such to get leveled and settled appropriately. After planting, to facilitate the proper establishment of plants, instant watering is done prior to the supply of regular irrigation (Wali et al. 2015).

Managing an *E. officinalis* orchard includes looking after the nutrient and water supply, maintenance of canopy architecture, field cleanliness, and taking plant protection measures on time (Pareek and Kitinoja 2011). Generally, young plants sprout with a specific degree of annual vegetative growth to develop an initial canopy. After two years, the plants build up a suitable canopy to bear fruits. However, for keeping up better growth, flowers and fruits should be removed in the first two years, and periodical watering, hoeing, weeding, plant protection, etc. should be performed accordingly. For young fruit-bearing orchard (2–7 years), trees need extra nutrients to continue desirable growth and fruiting. In fact, young trees exhibit profuse vegetative growth suppressing regular fruiting and, hence, need proper and judicious pruning preferably in the month of March–April, allowing the main branches to reach a height of 0.75–1 m from the ground. Eventually, only selected 4–6 branches are allowed to grow further (Wali et al. 2015).

#### Nutrient management

Application of a combination of organic and inorganic nutrients increases fruit production and quality; whereas, the use of vermicompost significantly improves fruit quality. The physical, biological and chemical properties of soil are influenced by these sources of nutrients. Dosage of manure and fertilizers differs based on soil fertility, age of the plant and frequency of fruiting. Usually, 10 kg FYM, 100 g nitrogen, 50 g phosphorus and 100 g potassium are applied to one-year-old plant. Annual increment of such dose should be assured up to 10 years, following which a stable dose is applied in the subsequent years. The complete dose of FYM, phosphorus, half of the nitrogen and MoP are applied around tree basins during December-January. The remaining half is applied in August. In any problematic land, 100–500 g boron, zinc sulfate and copper sulfate are supplemented along with regular fertilizers. Basal application of 100: 50: 50 g NPK/plant and 16 tons/ha FYM can also be used as well (Awasthi et al. 2009). Singh and Singh (2015a) suggested that the usage of synthetic auxins (α-naphthalene acetic acid; NAA) and gibberellic acid (GA_3_) in combination with thiourea during mid-May and mid-July may provide an effective solution to minimize yield losses caused by heavy fruit drop. This recommendation can be advocated in sodic soils characterized by production constraints such as limited availability of different mineral nutrients for optimum tree growth and yield. Plant growth regulators (PGRs) and various other nutrients play vital roles in improving the growth, development and quality of *E. officinalis* fruit. Foliar nutrient sprays are comparatively more effective for rapid absorption and utilization by plants, wherein soil pH is high and wide range of macro- and micro-elements are unavailable. Two foliar sprays of NAA (30 ppm) in well-established orchards during May–July improves fruit quality along with fruit retention (Yadav et al. 2010). As per the experimental findings of Singh et al. (2008), combined spraying of 0.5% ZnSO_4_ + 0.4% CuSO_4_ + 10 ppm NAA was found to be effective in improving plant growth and a simultaneous reduction in fruit drop. Foliar feeding of CuSO_4_ (0.4%) + MnSO_4_ (0.5%) + ZnSO_4_ (0.4%) twice during mid-May and mid-July is proven to be the best for improving physico-chemical attributes of *E. officinalis* fruits (Mishra et al. 2017a); whereas, foliar application of GA_3_ (150 ppm) was found to be the most effective to increase the vegetative growth and fruit yield (Mishra et al. 2017b). Similarly, foliar spray of boron and zinc showed an improved response in fruit yield. The highest fruit yield and quality (with increased vitamin-C content) per tree were recorded with the foliar sprays of 0.2% borax + 0.5% ZnSO_4_ (104.80 kg/tree) (Verma et al. 2008).

#### Water management

*E. officinalis* is being cultivated as a rain-fed tree and no irrigation is required in established orchards in normal soils especially during rainy and winter seasons. Only after manure and fertilizer application (during January–February) in the fruit-bearing plant, first irrigation should be given. However, the application of water should strictly be escaped at the flowering period (mid-March to mid-April). Basin system of irrigation is best suited for *E. officinalis*. Drip irrigation is also a promising practice and in water scarcity areas, pitcher irrigation is usually recommended for orchard establishment. During a typical dry period, significant stock girth and plant height can be recorded with the applications of a total number of 9 irrigations (based on IW/Pan-E ratio of 0.5) along with mulching, which eventually can save up to 20 cm of irrigation water (four irrigations) on a net area basis. So much so an additional area of orchard may be established with such saved water (Vashisht et al. 2018). Sometimes, fertigation in the place of sole irrigation or fertilizer application records a significant increase in the flowering frequency. Drip fertigation of 125% recommended dose of fertilizer (RDF) as water-soluble fertilizers (WSF) registered the highest values for the flower parameters (Suresh and Kumar 2014). The highest plant height, trunk girth and plant spread (east–west and north–south) can be registered by application of 125% RDF in the form of WSF via fertigation (Suresh et al. 2019).

#### Cropping system

The tree canopy of *E. officinalis* with sparse foliage facilitates abundant incoming daylight and assists intercropping within available spaces even under full-grown trees (Ghosh and Pal 2010), and thus, during initial 3–4 years of planting, such intercropping offers an exceptional prospect to make use of available interspaces in the orchard. Intercropping with turmeric, ginger and arbi, *E. officinalis* recorded promising results with respect to yield, available carbon, nitrogen, phosphorus and overall farm economics (Das et al. 2011). Apart from the above-mentioned plant species, *Amorphophallus* is another shade-preferring plant that can be commercially grown in *E. officinalis* orchard. Singh and Singh (2015b) demonstrated that growing elephant foot yam as an intercrop in *E. officinalis* plantation was found most to be suitable based on growth, yield and quality parameters (of both the crop), soil fertility status, gross income, net income, cost–benefit ratio, etc. In case of the arid region, winter crops like chickpea, cumin, fenugreek, and mustard, and rainy-season crop like moth bean are grown as intercrops with *E. officinalis* (Awasthi et al. 2009). Vegetables like bottle gourd, okra, coriander, cauliflower, pea, and turmeric; flowers like gladiolus and marigold have been found well suited for intercropping. In salt-affected or marginal soils, intercropping of spiny sesbania for few years is beneficial for amending the soil physico-chemical properties. Tuber crops can also be grown befittingly even under the dense shade of orchard (Singh and Singh 2015b). *E. officinalis* being a deep-rooted, deciduous tree with sparse foliage is proved to be a model plant for 2-, 3- or multi-tier cropping technique. Cropping system models such as *E. officinalis* with ber or guava (two-tier), or with phalsa (two-tier), or with spiny sesbania and wheat or barley, or with spiny sesbania and onion/garlic or brinjal, or with spiny sesbania and German chamomile (three-tier), etc. have been found much remunerative.

#### Fruit maturity, harvesting, and yield

Time of maturity of *E. officinalis* fruit is dependent upon desirable yield and processing quality. Commercial traits like days from flowering to maturity, heat units, color of fruit skin and total soluble sugar: acid ratio, etc*.* are taken into account during ascertaining the maturity index of any *E. officinalis* cultivar. At the maturity and ripening stage, the fruits initially become light green and then turn greenish-yellow or rarely brick red. Maximum ascorbic acid content is recorded in mature fruits, in contrast immature fruits are low in ascorbic acid and mineral content. During November–December, fruits are ready to be harvested via hand picking. Completely developed fruits are plucked (either in early- or in late-hours of a day) without any delay to avoid fruit dropping, particularly for ‘Banarasi’ and ‘Francis’ cultivars. A seedling tree takes 6–8 years to initiate fruit and a budded/grafted tree starts fruit bearing after 3 years of planting, but the latter may keep on fruiting up to 60–75 years of age (Pareek and Kitinoja 2011). An *E. officinalis* tree may bear 100–300 kg fruits /tree, yielding 15–20 tons/ha (Wali et al. 2015). The better yield of *E. officinalis* can be attained if better fruit retention along with other yield attributing characters is assured. Maholiya et al. (2015) reported the maximum fruit yield in cultivar ‘Kanchan’ (99.79 kg/tree) trailed by ‘Krishna’ (76.55 kg/tree). However, as high as ~ 220–280 kg per tree fruit yield may generally be recorded if proper agrotechnology is followed (Yadav et al. 2010). The mature fruits are usually very firm and, thus, facilitate large-scale harvesting, carriage and marketing even to distant regions (Pareek and Kitinoja 2011).

### Plant protection

#### Physiological disorders and their management

Chilling injury, necrosis, pink spots, and white specks are some major physiological disorders that affect the quality of *E. officinalis* fruits. Chilling injury results in the splitting of peel and sporadic ripening of fruits that eventually leads to decay. To avoid such injury, storage temperature should be optimized around 12 °C (Pareek 2010). During the hardening of endocarp, browning of innermost mesocarpic tissues along with epicarp results in the blackened fruit surface in the form of necrosis. In addition, owing to the deficiency of boron, random pink spots appeared on *E. officinalis* fruits that eventually deteriorate the fruit quality. To control both these disorders, spraying of borax (0.6%) thrice at two-week intervals (during September–October) is useful (Sharma 2006; Pareek 2010). White specks are the other major disorder that causes poor appearance and spongy texture of fruit at the curing and pickling stage. The frequency of white specks can be minimized via preservation of fruit segments in 0.04% K_2_S_2_O_5_ and 10% NaCl solution, followed by salting and dehydrating with 0.02% K_2_S_2_O_5_ and 10% NaCl after four weeks of storage (Premi et al. 1998).

#### Pathological disorders and their management

Major pathological diseases found in *E. officinalis* are rust, anthracnose, fruit rots, blue mold rots, etc. (Pareek and Kitinoja 2011), among which, rust is economically most important. Black pustules followed by ring pattern appearance on fruits are developed. *Revenelia emblica* Syd is the causal organism, which is an obligate parasite. Clean cultivation along with the removal of infected fruits and leaves (proper pruning) decreased infestation of this disease. Spraying 0.2% Zineb or 0.5% sulfur (wettable) three times from the month of July at four-week intervals proved to be effective for rust management. During August–September, *Colletotrichum* state of *Glomerella cingulata* results in anthracnose of fruits and leaflets. Dried up leaves initially appear that turn into dark brown smudges with the red margin and yellow halos (Pareek and Kitinoja 2011). Such infected plant parts should be amputated during inception of the disease. In addition, 0.1% Carbendazim or 0.2% Difolatan spray is recommended to get rid of anthracnose (Nallathambi et al. 2007; Wali et al. 2015). A pre-harvest fruit (Phoma) rot is usually observed frequently during the colder month of January. Such rot appears as small, lemon-colored lesions, which becomes enlarged and gets covered with funicles of conidiophores bearing spore (Mishra 1988; Wali et al. 2015). The disease usually resulted in a pre-mature fruit drop, which later mummified on the ground (Verma and Singh 2018). The disease can be characterized by necrotic spots, which extend to both ends of the fruit forming an eye-shaped spot. Multiple such lesions merge to create bigger pustules in heavily infested fruits (Nallathambi et al. 2007). Due to Phoma infection (by *Phoma exigna*), very quick decline in vitamin-C content is recorded in comparison to that of the gradual decline under storage condition (Reddy and Laxminarayana 1984). Other rots that are not so widespread are caused by *Aspergillus luchuensis* and *Fusarium acuminatum* (Sumbali and Badyal 1990). Dry rot sets off by *Cladosporium tenuissiumum* and *C. cladosporoids* is commonly seen in November and March, respectively. The initially colorless area along with a slightly soft spot appears that further turns into a round dark brown lesion (Jamaluddin 1978). In such circumstances, sodium hypochlorite (100–150 ppm) along with relevant anti-fungal agents should be applied depending on the source and degree of infestation (Pareek and Kitinoja 2011). Alternatively, pre-harvest dip of fruits at the rate of 4% borax or two sprays of 0.01% calcium nitrate with 0.1% Topsin M is efficient against fruit rot (Nath et al. 1992; Yadav and Singh 1999). Among the other pathological disorders, an infestation of *Penicillium islandicum* causes blue mold rot exhibiting brown patches with water-soaked areas. Further, three colors, i.e., bright yellow, purple-brown and bluish-green develop in heavy infestation. Yellow liquid exudes drops from the diseased patches; fruits exert bad odor and turn into a bluish-green or beaded look eventually. Nonetheless, proper fruit handling and good sanitary conditions during storage along with NCl_3_ and ozone gas treatment remains effective. In addition, fruit treatment with borax @ 0.5 g/l of water is promising as well (Wali et al. 2015).

#### Insect pests and their management

There is an array of insect pests from the order of Lepidoptera (*Betousa stylophora* Swinhae, *Celepa celtis*, *Gracillaria acidula, Indarbela tetraonis* and *Virachola Isocrates*), Homoptera (*Oxyrhachis tarandus*, *Nipaecoccus vastator*, and *Ceciaphis emblica*), Isoptera (*Odontotermes* spp*.*), and Cleoptera (*Myllocerus discolor*) (Pareek and Kitinoja 2011). Juvenile fruits are affected by fruit borers that feed on the developing seeds after laying of eggs and the subsequent emergence of caterpillars. During July–August, Endosulphan (0.05%) spray is effective against fruit borers, apart from the collection and destruction of affected fruits. As a precautionary measure, near *E. officinalis* orchards, pomegranate cultivation should be avoided (Pareek and Kitinoja 2011; Wali et al. 2015). The other category of the caterpillar is ‘bark-eating’ type (*Indarbela quadrinotata*, *I. tetraonis*) that causes up to 80% damage to the whole plant. The main trunk is affected and trunk tunnels are formed by this caterpillar. Feed on the bark under silken ribbon-shaped webs. Reduction of overcrowded branches and clean cultivation can manage this pest, but with an increasing infestation Furadan or Endrin spray (0.03%) during February–March or September–October is proven to be effective (Wali et al. 2015). Tender shoots are susceptible to shoot-gall maker (*Betanosa stylophora*). During the rainy season, young caterpillars bore into tender shoots and feed in pits. The damaged region develops gall formation. Shoot-gall maker attacks all the available varieties. The affected parts should be pruned and burnt to minimize the infestation. Iron or spoke can be inserted to kill the larvae or injecting Dichlorovas or Endosulphon @ 0.05% in the holes can equally be effective apart from the collection and mass destruction of gall affected shoots. Additionally, the application of 0.05% Monocrotophos in the month of July–August remains quite useful. Finally, aphids (*Schoutedenia emblica*) are gaining importance rapidly in the cultivation of *E. officinalis* as it infests tender shoots, leaves, flower bud and fruits. A single spray of 2% neem seed kernel extract, or 0.03% Dimethoate or 0.05% Phosalone at the initiation of new flush is efficient against aphids (Wali et al. 2015).

### In vitro propagation

**Fig. 5:**
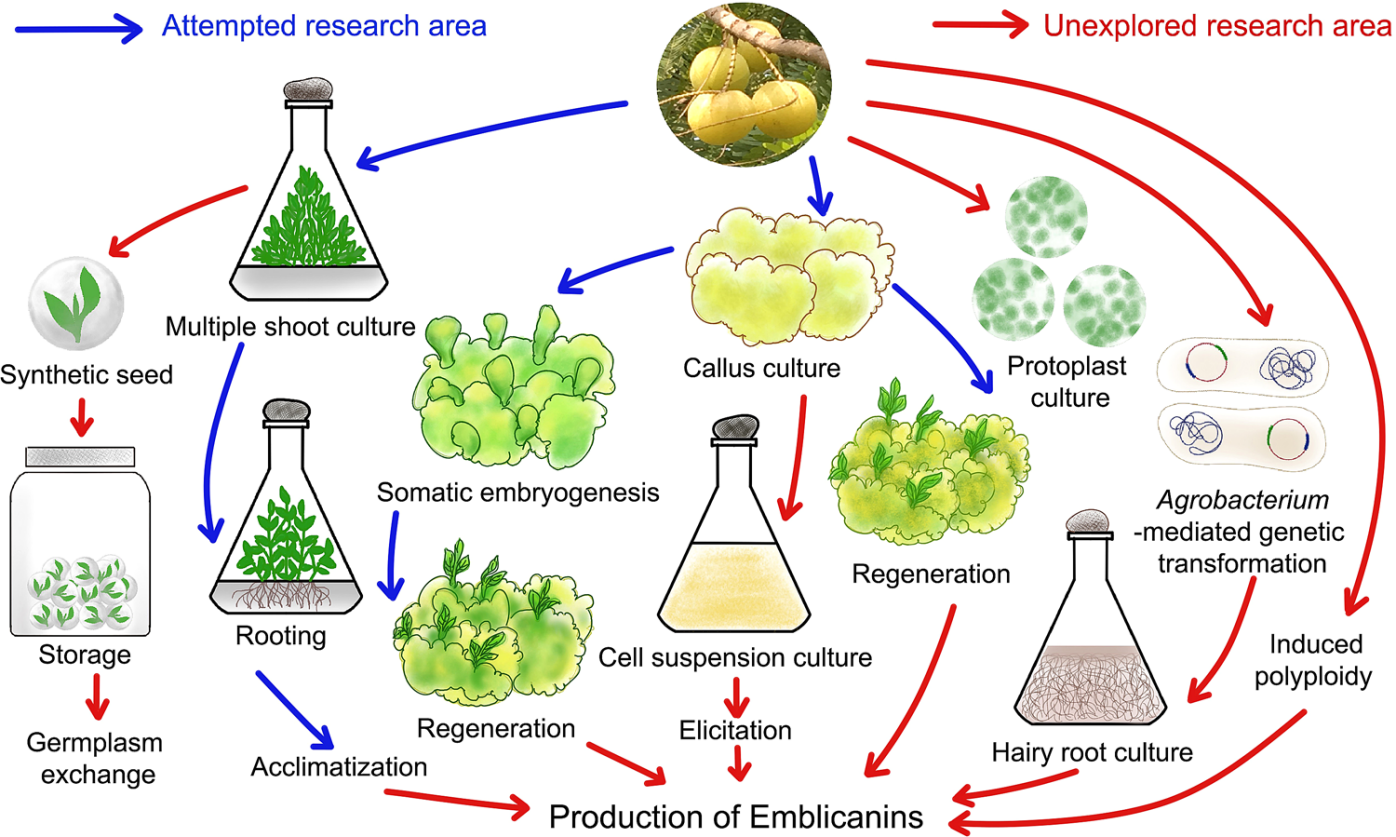
Diagram exhibiting the research areas that have been already studied and the areas that are yet to be explored regarding in vitro cell, tissue and organ culture of *Emblica officinalis* Gaertn. syn. *Phyllanthus emblica* L. (*Source*: unpublished photograph of Saikat Gantait)

In vitro propagation ensures the rapid multiplication of plantlets from plant cells and tissues on nutrient media under aseptic conditions (Mukherjee et al. 2019). Conventionally, *E. officinalis* is propagated through seeds and asexually by budding and grafting. Propagation through seeds is not beneficial since seeds possess dormancy and do not produce true-to-type plants owing to cross-pollination and seed-derived plants bear inferior quality fruits (Mishra et al. 2011). To overcome this issue, micropropagation techniques were employed to produce large-scale true-to-type and disease-free plants. Several methods of in vitro propagation have been executed in *E. officinalis* to date. This review compiles different approaches attempted for micropropagation and developmental work done in *E. officinalis* (Fig. 5).

#### Selection of explant

A proper selection of explant is important in any micropropagation study. The stage, regenerating ability and preferably disease-free were taken into consideration while selecting explant (Mukherjee et al. 2019). A number of explants such as nodal segments, shoots, hypocotyls, epicotyls, embryo, root–shoot node, and leaf have been used by researchers for both direct and indirect regeneration in *E. officinalis* (Table 3). Nodal segment and shoots are generally used for direct organogenesis (Verma and Kant 1999; Mishra and Pathak 2001; Mishra et al. 2006; Goyal and Bhadauria 2008; Patidar et al. 2010). Preferably, the 10–15th nodal segment portion was taken as they gave better response than the young nodal segment because they cannot withstand the disinfection process and older segment showed lower response because of mature tissues (Mishra and Pathak 2001). For indirect organogenesis via callus formation, cotyledons, hypocotyls, epicotyls, embryo, and leaf are being used (Sehgal and Khurana 1985; Gupta et al. 1994; Verma and Kant 1999; Al-Sabah et al. 2012; Priyanka et al. 2014; Thilaga et al. 2013; Priyanka and Singh 2015). Hypocotyls, epicotyls, and zygotic embryos are found to be more promising than leaf for indirect somatic embryogenesis. Other explants such as root and root shoot nodes were also reported to be used (Gour and Kant 2009).

**Table 3:** Factors influencing in vitro regeneration of *Emblica officinalis* Gaertn. syn. *Phyllanthus emblica* L

Explant	Surface disinfection	Basal media	Carbon source	Solidifying agent	PGR (type and conc., mg/l or μM*)	Additives (mg/l)	Culture condition [T; LI (lux or PPFD^#^); PP; RH]	Response	References
Endosperm	2% Cetrimide → chlorine water 5 min	MS	2% sucrose	0.8% agar	1 2,4-d + 1 Kin	NM	25 ± 2 °C; 1000–2000; NM; 55 ± 5%	Active callus development	Sehgal and Khurana (1985)
					0.2 BAP + 0.1 IAA			Shoot regeneration from callus	
		Liquid MS			0.002 NAA			Rooting	
Hypocotyl	0.1% HgCl_2_ 5–7 min	MS	NM	NM	2 2,4-D + 0.5 NAA	50 AA + 50 CA + 100 PVP + 500 AC	28 ± 2 °C; 2500–3000; 16 h; 40–50%	Callus formation	Gupta et al. (1994)
					3 BAP + 2 Kin + 0.1 2,4-D			Differentiation of calli into shoot buds	
Nodal segment, cotyledons, cotyledonary nodes, hypocotyls	2% Extran → 0.1% HgCl_2_	MS	NM	0.8% agar	5 BAP + 0.5 NAA	25 AdS + 25 AA + 25 CA + 25 PVP	26 ± 2 °C; 2000; 16 h; 50–60%	Multiple shoot	Verma and Kant (1999)
					2 2,4-D	25 AA		Callus induction	
					2 BAP or 2 Kn	25 AdS		Callus differentiation into shoot buds	
		½MS	1.5% sucrose		2 IBA			60–80% rooting	
Axillary/ nodal shoot	NM	Modified MS	3% sucrose	0.8% agar	0.4 Kn + 1 GA_3_	NM	25 ± 2 °C; 2000; 16 h; 50–55%	Multiple shoot	Mishra and Pathak (2001)
Shoots	0.1% Bavistin + 60,750 µM Rifampicin + Tween-20 1 h → 0.1% HgCl_2_ 8 min	MS	3% sucrose	0.8% agar	4330* GA_3_ + 13,900* Kn + 342,110* Glutamine	NM		Multiple shoot	Mishra et al. (2006)
		½MS			49,200* IBA + 10,740* NAA			Rooting	
Nodal segment	Tween-20 5–10 min → 0.5–1.0% Bavistin 30 min → AA + CA + PVP 15–30 min → 0.1–1% (streptomycin + chloramphenicol) 30 min → 4–5% hypo solution + 0.1–1% HgCl_2_	MS	NM	NM	4.44* BAP + 2.46* IBA	NM	25 ± 2 °C; NM; 16 h; NM	Multiple shoot	Goyal and Bhadauria (2008)
Root, shoot nodes	0.1% HgCl_2_ 5 min	MS	NM	NM	2 BAP	NM	26 ± 2 °C; 2000; 16 h; NM	Multiple shoot	Gour and Kant (2009)
		½MS			2 IBA			Rooting	
Nodal segment	2% tween 20 10 min → 70% ethanol 1 min → 1% Bavistin Ca(OCl)_2_ → 0.2% HgCl_2_ 10 min	MS	3% sucrose	0.75% agar	4 BAP + 0.5 NAA	NM	25 ± 2 °C; 1200; 16 h; 70%	Multiple shoot	Patidar et al. (2010)
					2 IBA + 0.5 BA			Rooting	
Epicotyl	5% Labolene → 0.1% Bavistin 10 min → 0.1% HgCl_2_ 5 min	MS	3% sucrose	0.8% agar	8.8* BA + 1.425* IAA	NM	25 ± 2 °C; 40; 16 h; 55–60%	Multiple shoot	Nayak et al. (2010)
		½ MS			14.7* IBA			Rooting	
Cotyledon, hypocotyl, epicotyl, leaf	30% Chlorax + a drop tween-20 15 min	MS	1.5% sucrose	0.15% phytagel	1 2,4-d + 0.1 Kn	NM	25 ± 2 °C; 1000; 16 h; NM	Somatic embryogenesis	Al-Sabah et al. (2012)
		½ MS			1 IBA			Adventitious roots	
Seeds	1% Bavistin for 30 min → 2–3 drops Tween 20 for 10 min → 70% ethanol 5 min→ NaCl 10 min → HgCl_2_ 5 min	MS	NM	NM	1 Pic	NM	NM	Callus induction	Madharia and Dutta (2012)
					1 BAP			Shoot and root initiation	
Cotyledon	NM	MS	NM	NM	1 2,4-d	NaCl	25 ± 2 °C; 1400; NM; NM	Callus induction	Priyanka et al. (2014)
Zygotic embryo, Leaf	0.1% HgCl_2_ 15 min	MS	3% sucrose	0.8% agar	0.45* 2,4-d + 22* BAP		25 ± 1 °C; 40^#^; 16 h; NM	Callus induction	Thilaga et al. (2013)
					0.45* 2,4-d + 11* BAP			33.8% somatic embryogenesis	
Cotyledon	NM	MS	3% sucrose	NM	2,4-d	NM	25 ± 2 °C; 1400; NM; NM	Callus induction	Priyanka and Singh (2015**)**
					NAA			Callus induction, somatic embryogenesis	
					IAA, IBA			Somatic embryogenesis	
					2,4-d + Kn			Somatic embryogenesis with callus formation	

2,4-d, 2,4 dichlorophenoxyacetic acid; AA, ascorbic acid; AC, activated charcoal; AdS, adenine sulfate; BA, *N*^6^-benzyladenine; BAP, *N*^6^-benzylaminopurine; CA, citric acid; GA_3,_ gibberellic acid; IAA, indole-3-acetic acid; IBA, indole-3-butyric acid; Kn, kinetin; LI, light intensity; MS, Murashige and Skoog; NAA, α-naphthalene acetic acid; NM, not mentioned; PGR, plant growth regulator; Pic, picloram; PP, photoperiod; PVP, polyvinylpyrrolidone; RH, relative humidity; *T*, temperature

#### Procedures for disinfection of explants

After selection of explant, surface disinfection of the explant to reduce contamination is the next determining step. The concentration and duration of disinfection are influenced by the type and stage of explant; otherwise, they may exert some unfavorable influence on the growth and development of the explant. In the case of *E. officinalis*, 2–3 drops of Tween-20 for 5–10 min, subsequently 0.1% (*w/v*) mercuric chloride (HgCl_2_) treatment for 5 min with successive washing with sterile water was commonly followed. Among the array of explants used, nodal segment proved an explant of choice owing to their freshness (and greenish nature) even following HgCl_2_ treatment but at the cost of latent contamination and phenolic leaching. Most of shoot cultures of *E. officinalis* showed higher fungal infection with low bacterial contamination (Goyal and Bhadauria 2008). Reports have mentioned that the survival percentage of explants increases when they were dipped in HgCl_2_ for lesser time. On the other hand, an increase in concentration (0.5% instead of 0.1%) and duration of HgCl_2_ treatment resulted in browning and eventual death of explants, and in such situation, sodium hypochloride solution appeared to be a better alternative to control the infection rate (up to 90%) (Goyal and Bhadauria 2008). Yet, the use of HgCl_2_ was quite frequent and remained most effective controller of contaminations during in vitro establishment of *E. officinalis*. Some the researchers used 2% (*v/v*) extran (Verma and Kant 1999) and 5% labolene (Nayak et al. 2010) instead of Tween 20 prior to treating with HgCl_2_. Some have used 0.1–1.0% (*w/v*) Bavistin either before or after Tween 20 treatment. In addition, the use of freshly prepared chlorine water for endosperm treatment prior to its inoculation was reported too (Sehgal and Khurana 1985). One report cited the use of antioxidants such as ascorbic acid, citric acid and polyvinylpyrrolidone (PVP) for explant treatment before inoculation to check the release of phenolic compounds (Goyal and Bhadauria 2008).

#### Basal nutrient medium

The basal medium serves as the source of both macronutrients and micronutrients to the explant under in vitro condition (Gantait and Panigrahi 2018). It also provides vitamins and other organic components required for the growth and development of plants. Almost all researchers have used Murashige and Skoog (MS) (Murashige and Skoog 1962) medium for various experiments in *E. officinalis* (Sehgal and Khurana 1985; Gupta et al. 1994; Verma and Kant 1999; Mishra and Pathak 2001; Mishra et al. 2006; Goyal and Bhadauria 2008; Gour and Kant 2009; Patidar et al. 2010; Nayak et al. 2010; Al-Sabah et al. 2012; Madharia and Dutta 2012; Priyanka et al. 2014; Thilaga et al. 2013; Priyanka and Singh 2015). Many have reported that half-strength MS medium was effective for rooting in *E. officinalis* (Verma and Kant 1999; Mishra et al. 2006; Gour and Kant 2009; Nayak et al. 2010; Al-Sabah et al. 2012). The use of liquid MS medium in full-strength was found to be effective especially during the root induction of in vitro shoots (Sehgal and Khurana 1985). In some instances, Gamborg’s B5 medium (Gamborg et al. 1968) and woody plant medium (WPM) (Lloyd and McCown 1981) were also used by Goyal and Bhadauria (2008), but found incomparable to the promising results of MS medium; nonetheless, least amount of phenol leaching was found in WPM.

#### Carbon source

Carbon is one of the major constituents of the living cells and plays an important role in plant metabolism. It plays a major role in energy production for the functioning of plant cells and also acts as an osmotic regulator in the nutrient medium (Gantait and Kundu 2017). Sucrose was commonly used as the carbon source in the nutrient medium but the concentration may vary. Generally, 3% (*w/v*) sucrose was used by most of the researchers (Mishra and Pathak 2001; Mishra et al. 2006; Patidar et al. 2010; Nayak et al. 2010; Thilaga et al. 2013; Priyanka and Singh 2015). For two instances, 2% or even as low as 1.5% sucrose was used for in vitro regeneration of multiple shoots (Sehgal and Khurana 1985; Al-Sabah et al. 2012). In another instance, 1.5% sucrose was used for rooting of regenerated shoots (Verma and Kant 1999). However, the use of table sugar instead of laboratory-grade sucrose for cost reduction was reported to be useful too; and it was also reported that a higher number of shoot and root induction, and increased root length of *E. officinalis* were recorded in medium containing table sugar rather than other alternatives (Gour and Kant 2011).

#### Physical conditions of growth room

Physical conditions for instance, temperature, light intensity, photoperiod, and relative humidity play a major role in the growth and development of explant during micropropagation. They have to be artificially controlled according to the requirement of the species. Temperature plays a vital role in the metabolic activities of the cells. For in vitro regeneration of *E. officinalis*, the temperature was maintained at 25 ± 2 °C as cited in most of the reports (Sehgal and Khurana 1985; Mishra and Pathak 2001; Goyal and Bhadauria 2008; Patidar et al. 2010; Nayak et al. 2010; Al-Sabah et al. 2012; Priyanka et al. 2014; Thilaga et al. 2013; Priyanka and Singh 2015). However, maintenance of in vitro cultures at a temperature of as high as 28 ± 2 °C was also reported (Gupta et al. 1994). Light intensity and photoperiod are necessary for the plant, as light is required to carry out photosynthesis. From the compiled reports, it was noted that cultures of *E. officinalis* were maintained under a photoperiod of 16 h (Gupta et al. 1994; Verma and Kant 1999; Mishra and Pathak 2001; Goyal and Bhadauria 2008; Gour and Kant 2009; Patidar et al. 2010; Nayak et al. 2010; Al-Sabah et al. 2012; Thilaga et al. 2013) but the intensity of light varies from 1000 to 3000 lx or maintained at 40 μmol/m^2^/s (Nayak et al. 2010; Thilaga et al. 2013). Maintaining relative humidity (RH) of the culture room is also vital as high relative humidity can result in hyperhydricity of cells and contamination in the cultures (Gantait and Kundu 2017). Cultures of *E. officinalis* are maintained at 50–60% RH (Sehgal and Khurana 1985; Verma and Kant 1999; Mishra and Pathak 2001; Nayak et al. 2010); whereas, maintenance of cultures at RHs of < 45% or > 65% was also reported (Gupta et al. 1994; Patidar et al. 2010).

#### Plant growth regulators

Along with the basal medium and carbon source, PGRs play a major role in the development of plant cells. Major plant growth regulators are auxin, gibberellin and cytokinin. Cytokinin and auxin ratio determines the shoot and root development as well as callus formation. Gibberellin is generally used to break dormancy and initiate seed germination. Use of different PGRs by various researchers for in vitro propagation of *E. officinalis* has been summarized (Table 3).

#### Direct regeneration

Direct regeneration of shoots and roots is achieved by supplementing the culture medium with cytokinin and auxin. Generally higher cytokinin than auxin promotes shoot development and higher auxin than cytokinin promotes rooting. Different cytokinins like N^6^-benzylaminopurine (BAP) and Kinetin (Kn) have been reported for shoot regeneration but BAP stands out to be most used and gives promising results when used alone or with auxin. Enriching the media with BAP (1–4 mg/l) alone for shoot proliferation was reported by a number of researchers (Verma and Kant 1999; Gour and Kant 2009; Madharia and Dutta 2012). Various combinations for multiple shoot proliferation have also been reported in *E. officinalis*, such as, BAP + NAA (Verma and Kant 1999; Patidar et al. 2010), BAP + indole-3-butyric acid (IBA) (Goyal and Bhadauria 2008), N^6^-benzyladenine (BA) + indole-3-acetic acid (IAA) (Nayak et al. 2010). Combination of Kn and GA_3_ has been reported (Mishra and Pathak 2001; Mishra et al. 2006) for multiple shoot generation. For regeneration of roots, use of various auxins such as NAA and IBA was reported but in majority of the cases, IBA was supplemented alone (Verma and Kant 1999; Gour and Kant 2009; Nayak et al. 2010; Al-Sabah et al. 2012). Combination of IBA with NAA or sole application of NAA for rooting was reported in fewer instances (Sehgal and Khurana 1985; Mishra et al. 2006). Various additives such as ascorbic acid, citric acid and PVP were also supplemented in the media to check the leaching of phenolic compounds (Gupta et al. 1994; Verma and Kant 1999).

#### Callus-mediated regeneration

*Callus induction*: A callus is an unorganized and unspecialized mass of tissue as a result of a wound on the plant surface. When a basal medium is supplemented with appropriate PGRs, it gives rise to callus, which further differentiated into whole plantlets (Mitra et al. 2020). From the compiled reports on in vitro propagation of *E. officinalis*, it was observed that when endosperm, hypocotyls, epicotyls, embryo, and leaf were used as explant and inoculated in sole 2,4 dichlorophenoxyacetic acid (2,4-d) enriched medium (Gupta et al. 1994; Verma and Kant 1999; Priyanka et al. 2014; Priyanka and Singh 2015), the explants induced calli and further differentiated into shoot and root buds. However, the addition of BAP with 2,4-d (0.45 µM 2,4-d + 22 µM BAP) was reported to induce calli from zygotic embryo and leaf explants (Thilaga et al. 2013). Use of 1 mg/l picloram (Pic)-supplemented media as an alternative of 2,4-d was effective during induction and proliferation of embryogenic calli (Madharia and Dutta 2012). Similarly, callus was induced from endosperm when the same was inoculated in 2,4-d-free medium fortified by 0.2 mg/l BAP and 0.1 mg/l IAA (Sehgal and Khurana 1985). From the concerned reports, it was evident that calli were induced mainly in 2,4-d-enriched media but there were some cases wherein Pic or BAP:IAA was useful.

*Regeneration from callus* When the calli were transferred into cytokinin and auxin-rich media, they were differentiated into shoots and roots, respectively. When they were subcultured on media enriched with either BAP or Kin or both, induction of shoot buds were observed. For instance, in 0.2 mg/l BAP + 0.1 mg/l IAA, shoot regeneration was reported but failed to initiate roots. However, rooting of regenerated shoots was achieved in liquid MS medium supplemented with 0.002 mg/l NAA (Sehgal and Khurana 1985) or MS medium with 2 mg/l IBA, resulting in 60–80% of rooting (Verma and Kant 1999). Higher concentration of BAP (1–3 mg/l BAP) was also reported to be effective in differentiation of callus into shoots (Verma and Kant 1999; Madharia and Dutta 2012). BAP along with Kin (3 mg/l BAP + 2 mg/l Kin + 0.1 2,4-d) was used for more effective shoot formation from callus (Gupta et al. 1994).

#### Somatic embryogenesis

*Somatic embryo induction *Somatic embryogenesis is the development of an embryo from somatic cells of the plant under suitable conditions. It plays a vital role in woody plants for clonal propagation, cryopreservation, and can also be beneficial towards synthetic seed production for germplasm exchange (Gantait et al. 2018). Somatic embryogenesis (direct as well as indirect via. callus) was reported using basal MS media fortified by 1 mg/l 2,4-d and 0.1 mg/l Kn which further develops to shoot and root (Al-Sabah et al. 2012). However, as high as 33.8% somatic embryogenesis was obtained on MS media fortified by 0.45 µM 2,4-d and 11 µM BAP (Thilaga et al. 2013). In that report, a number of developmental stages were exhibited when embryogenic callus along with somatic embryos were cross-sectioned and studied. Most of the somatic embryos appeared as cup-like structure at the peripheral region of calli. Direct somatic embryogenesis was obtained in media enriched with IAA and IBA and indirect via callus was obtained in MS media fortified with 2,4-d and Kn (Priyanka and Singh 2015). From the above reports it is evident that 2,4-d-enriched media resulted in somatic embryogenesis in *E. officinalis* in the majority of the instances.

*Regeneration from somatic embryos *To date, there are three reports on regeneration from somatic embryogenesis in *E. officinalis*. Regeneration of plantlets from somatic embryos was seen in PGR-free MS medium (Al-Sabah et al. 2012; Priyanka and Singh 2015) but only one report cited the use of ABA and Kin (3.78 µM ABA or 0.46 µM Kin) for regeneration from somatic embryos (Thilaga et al. 2013). However, no specific stage of somatic embryo was mentioned that resulted in successful regeneration.

### Acclimatization

Acclimatization involves the transfer of in vitro plantlets to the soil and evaluates the survival percentage of the micropropagated plants. The ultimate aim always remains to assess the performance of the in vitro plants in the natural environment. A handful of reports are there on the acclimatization of *E. officinalis* plantlets in various combinations of soil, sand and compost. Acclimatization of plantlets was successfully achieved in autoclaved sand + soil + farmyard manure mixed either in 1:1:1 (*v/v*) ratio or in 6:2:1 (Mishra et al. 2006; Thilaga et al. 2013). Sand + peatmoss + humus mixed in 1:1:1 ratio along with spraying of Bavistin® was also effective for this purpose (Al-Sabah et al. 2012). The use of sterile cocopeat mixed with Bavistin® solution (Madharia and Dutta 2012) or simple soil + soilrite (3:1) mixture (Verma and Kant 1999) were sufficient to exert significant survival rate during acclimatization. Likewise, as high as 80% survival rate of acclimatizing plantlets was reported even in ordinary garden soil (Nayak et al. 2010).

### Production of secondary metabolites from plant parts

Secondary metabolites are compounds that are not involved in the growth and development of a plant but are involved in the interaction of plant with its environment (Pagare et al. 2015). Such compounds generally provide protection against biotic and abiotic stresses. Some are even used as drugs, dyes, flavors, etc. that increase their economic value. In *E. officinalis*, secondary metabolites in the form of phenolic compounds are responsible for its antioxidant properties and were estimated to be 439.9 mg/g of fruit powder when the ethyl acetate fraction was subjected to Sephadex LH-20 chromatography and reverse-phase HPLC (Liu et al. 2008). Another report cited the total phenolic content of ethanol extract of *E. officinalis* fruit to be 362.43 ± 11.22 mg GAE/g (Gallic acid equivalent) and total flavonoid content to be 6.40 ± 0.88 mg QE/g (quercetin equivalent) (Pientaweeratch et al. 2016). In fresh fruit aqueous extract of *E. officinalis*, the total phenolic content was determined to be 59.18 ± 2.91 mg GAE/g by Folin–Ciocalteu assay, total flavonoid content was estimated to be 38.50 ± 2.84 mg CE/g (catechin equivalent) by aluminum chloride colorimetric assay and total tannin content was estimated to be 44.28 ± 3.09 mg TAE/g (tannic acid equivalent) using spectrophotometry (Gunti et al. 2019). However, there is scanty of reports on production or extraction of secondary metabolites from in vitro culture of *E. officinalis*, wherein such plant tissue culture-based approaches might be quite useful to enhance the production of desirable secondary metabolites (emblicanin-A and -B or tannins in this case) of pharmaceutical importance.

### Molecular marker-based biotechnological interventions

The majority of studies on molecular markers in *E. officinalis*, to date, mainly focused on random amplified polymorphic DNA (RAPD), which is a widely adopted method in DNA fingerprinting investigation for molecular taxonomy, genotypic differentiation, and other applications (Table 4). Dnyaneshwar et al. (2006) have studied eleven cultivars of *E. officinalis* for the identification of their species on the basis of RAPD-sequence characterized amplification region (SCAR) markers. Bharmauria et al. (2010) developed an effective method for DNA isolation from leaf samples of eight *E. officinalis*. The authors used a previously published protocol by Warude et al (2003) with a slight modification of PVP amount (120 mg) per gram of leaf samples which increased the amount of DNA with good quality; they further concluded that the protocol could be useful for polymorphism and genetic variation studies in *E. officinalis* or related plants species which is having a high amount of polyphenols and polysaccharides (Bharmauria et al. 2010). The RAPD patterns were obtained from seven varieties of *E. officinalis* by Chaurasia et al. (2009) and these data could serve as an important input to the traditional method of identification of species variation that only based on morphological traits. A DNA isolation protocol had been developed for genetic fingerprinting studies by Nagarajan et al. (2011) with modification of cetyltrimethylammonium bromide (CTAB) protocol. In this study, authors kept pH stable during all the steps of DNA isolation by adding NaOH and finally high quality of DNA was achieved from leaf samples of *E. officinalis*. Five microsatellites were identified as polymorphic out of six markers developed in *E. officinalis*. These five microsatellites could be useful for the identification of clones, reproductive biology, and genetic structure in this plant species (Pandey and Changtragoon 2012). It has been reported that *E. officinalis* germplasm showed that remarkably high genetic variability and further reported that putative quantitative trait locus (QTLs) could be used to develop new or novel plant varieties (Mawalagedera et al. 2014). Eight different varieties of *E. officinalis* were investigated by Singh et al. (2014) to extent of genetic variability and relationship between different plant species based on RADP and rDNA polymorphism for breeding programs. Khomdram et al. (2014) optimized genomic DNA isolation using the CTAB extraction method and PCR amplification of DNA, for nineteen wild fruit species including *E. officinalis*. Kumar et al. (2016) reported a first time transcript associated with each gene involved in vitamin-C and flavonoid biosynthesis in *E. officinalis,* and further authors concluded that this important research could be useful for future functional genomics and molecular studies. Thilaga et al. (2017) developed RAPD markers for discrimination of susceptible and tolerant genotypes of *E. officinalis* against shoot-gall marker (*Betousa stylophora* swinhoe). Seven related species of *E. officinalis* were evaluated for their genetic diversity using microsatellite markers. The authors further concluded that fifteen markers, which were developed from their study, could be useful for the assessment of genetic variability, gene flow, and population genetic structure of *E. officinalis* or related plant species (Geethika et al. 2018). In another study, Liu et al. (2018) developed expressed sequence tag-simple sequence repeat (EST-SSR) markers (twenty highly polymorphic) for *E. officinalis* to investigate the gene flow and population genetic structures. The above studies have shown that all the markers developed and investigated for crop improvement and breeding program, however, there is still a wide scope for more intensive studies on the development of reproducible molecular markers (like SCoT, AFLP, SNP, RAMP, etc*.*) and characterization of *E. officinalis* germplasms and subsequent their genetic improvement against biotic and abiotic stresses.

**Table 4:** Application of molecular marker-based approaches on *Emblica officinalis* Gaertn. syn. *Phyllanthus emblica* L

Plant Samples	DNA/RNA isolation methods	Primer sequences and/or accession numbers	PCR conditions	Key findings	References
Leaf tissue	Modified DNA isolation protocol of Warude et al. (2003)	OPA-9 (5′GGGTAACGCC3′), OPA-14 (5′TCTGTGCTGG3′)	Initial denaturation at 94 °C for 5 min; 45 cycles at 94 °C for 1 min (denaturation), 36 °C for 1 min (annealing), and 72 °C for 2 min (extension); final extension at 72 °C for 5 min	Improved isolation of DNA samples with high purity and quantity	Bharmauria et al. (2010)
Young leaves	CTAB method with slight modification	55 random primers of different groups	Hot-start at 94 °C for 2 min; 40 cycles at 94 °C for 30 s (denaturation), 42 °C for 1 min (annealing), and 72 °C for 2 min (extension); final extension at 72 °C for 8 min	Distinguish closely related varieties based on their RAPD banding patterns	Chaurasia et al. (2009)
Fresh leaf tissues	CTAB	5′CAGATCTCGTGTAAAAAGCGTTG3′, 5′TGCAGTGAATTCCAAGTGTTTC3′	Initial denaturation at 94 °C for 5 min; 45 cycles at 94 °C for 1 min (denaturation), 36 °C for 1 min (annealing), and 72 °C for 2 min (extension); final extension at 72 °C for 5 min	SCAR marker was found useful for identification of genotypes	Dnyaneshwar et al. (2006)
Young leaves	DNeasyPlant Mini kit	SNX-F: GTTTAAGGCCTAGCTAGCAGAATC SNX-R: ATTCTGCTAGCTAGGCCTTAAACAAAA	Initial denaturation at 95 °C for 5 min; 38 cycles of 30 s at 94 °C (denaturation), 45 s at primer specific 47.7–52.8 °C (annealing), and 45 s at 72 °C (extension); final extension at 72 °C for 10 min	Developed microsatellite markers could be used to study the population genetic structure, gene flow and genetic diversity	Geethika et al. (2018)
Young leaf tissue	RNA was extracted using the protocol described by Kumar and Singh (2012)	PE21382, PE17828, PE17379, PE15252, PE14485, PE14389, PE14171, PE11297, PE10572, PE10156, PE9600, PE8480, PE8467, PE7779, PE7362, PE6950, PE6781, PE4618, PE788, and PE399	Initial denaturation at 94 °C for 5 min; 30 cycles at 94 °C for 30 s (denaturation), at 57 °C to 63 °C (locus specific) for 30 s (annealing), and at 72 °C for 30 s (extension); final extension at 72 °C for 5 min	Markers will be valuable for studying the population genetics and for mining genes	Liu et al. (2018)
Young tender leaves	DNeasy Plant mini kit	SCAR (F: CAGATCTCGTGTAAAAAGCGTTG; R: TGCAGTGAATTCCAAGTGTTTC), Phyll112 (F: TCGCTTTTATTTTCTTCAGTTCC; R: AAACCCACTGAGCATGAACC), Phyll68 (F: CAGGGACATTACACGGACAAC; R: CAGCCTAAGACAACTCTCATTTACC), Phyll53 (F: CTTTCTCCAGCCACCAAATG; R: GTTGGTGGGTTTTCAACCTG), Phyll31 (F: AACTGGTGACTCCCCTTTACTC; R: TCCTTGGCTGAATTTTGGAG), Phyll13 (F: AAGATCCGGCTTTAAAACTTTG; R: GCTAGCACTCTTCCTTCTTGC), Phyll7 (F: CGGGAAAGAGAAACGAAATG; R: GCATCAGGTGGACTTCTTGG)	Initial denaturation at 94 °C 10 min; 35 cycles of 20 s at 94 °C (denaturation), 20 s at 48 °C (annealing), and 2 min at 72 °C (extension); final extension at 72 °C for 10 min	Information can be used to develop genetically superior varieties	Mawalagedera et al. (2014)
Leaves	CTAB	A49325 (5 ´CGAAATCGGTAGACGCTACG3´), A49865 (5´GGGGATAGAGGGACTTGAAC3´)	Initial denaturation at 94 °C for 5 min; 40 cycles of 94 °C for 1 min (denaturation), 36 °C for 1 min (annealing), and 72 °C for 2 min (extension); final extension at 72 °C for 5 min	Yielding high-quality intact DNA for genetic fingerprinting as well as for amplification of chloroplast genes for molecular analysis	Nagarajan et al. (2011)
Young leaf	DNEasy Plant Minikit	21-mer (5′CTCTTGCTTACGCGTGGACTA3′), 25-mer (5′TAGTCCACGCGTAAGCAAGAGCACA3′)	Initial denaturation at 94 °C for 3 min; 30 cycles at 94 °C for 45 s (denaturation), 50 °C to 67 °C for 30 s (annealing), and at 72 °C for 1.5 min (extension); final extension at 72 °C for 10 min	Application in population genetics, they can also be used for clone and provenance identification	Pandey and Changtragoon (2012)
Fresh leaves	CTAB	ITS-1 (5′TCCGTAGGTGAACCTGCGG 3′), ITS-4 (5′TCCTCCGCTTATTGATATGC3′)	Initial denaturation at 94 °C for 3 min; 36 amplification cycles of 94 °C for 40 s (denaturation), 50 °C for 40 s (annealing), and 72 °C for 2 min (extension); final extension at 72 °C for 7 min	ITS regions as DNA barcode at higher levels can serve as an additional approach for identification and genetic cataloging germplasms for crop improvement	Singh et al. (2014)
Fresh leaves	CTAB	Forward 5 AGCGAGTCTTCATAGGGCGATTGT 3; Reverse 5TAGCTCTGGGTTCGAGTGGCATTT 3	95 °C, 3 min; 35 cycles at 94 °C, 50 s; 68 °C, 50 s; 72 °C, 2 min; and a final extension at 72 °C for 10 min	High-quality DNA isolation, amplification using housekeeping Actin gene	Khomdram et al. (2014)
Young leaves	Total RNA was isolated using the method described by Kumar and Singh (2012)	–	–	Transcripts containing SSR markers were indeed abundant	Kumar et al. (2016)
Leaf tissue	CTAB	OPAG15; OPAR10; OPAS09; OPAL12; OPU06; OPN02; OPBH04; OPAJ14; OPO14; OPAP20; OPAX06; OPF14; OPC11; OPAD15; OPO09; OPK05; OPI08; OPS10; OPP20; OPAN05	Initial denaturation at 94 °C for 4 min; 94 °C for 1 min (denaturation); 30 cycles (annealing) at 37 °C for 1 min, and 72 °C for 2 min (extension); final extension at 72 °C for 6 min	A reliable method has been developed for discrimination of tolerant and susceptible genotypes	Thilaga et al. (2017)

CTAB, cetyltrimethylammonium bromide; ITS, internal transcribed spacer; PCR, polymerase chain reaction; RAPD, random amplified polymorphic DNA; SCAR, sequence characterized amplified region

### Nanotechnology: green synthesis of nanoparticles

**Fig. 6:**
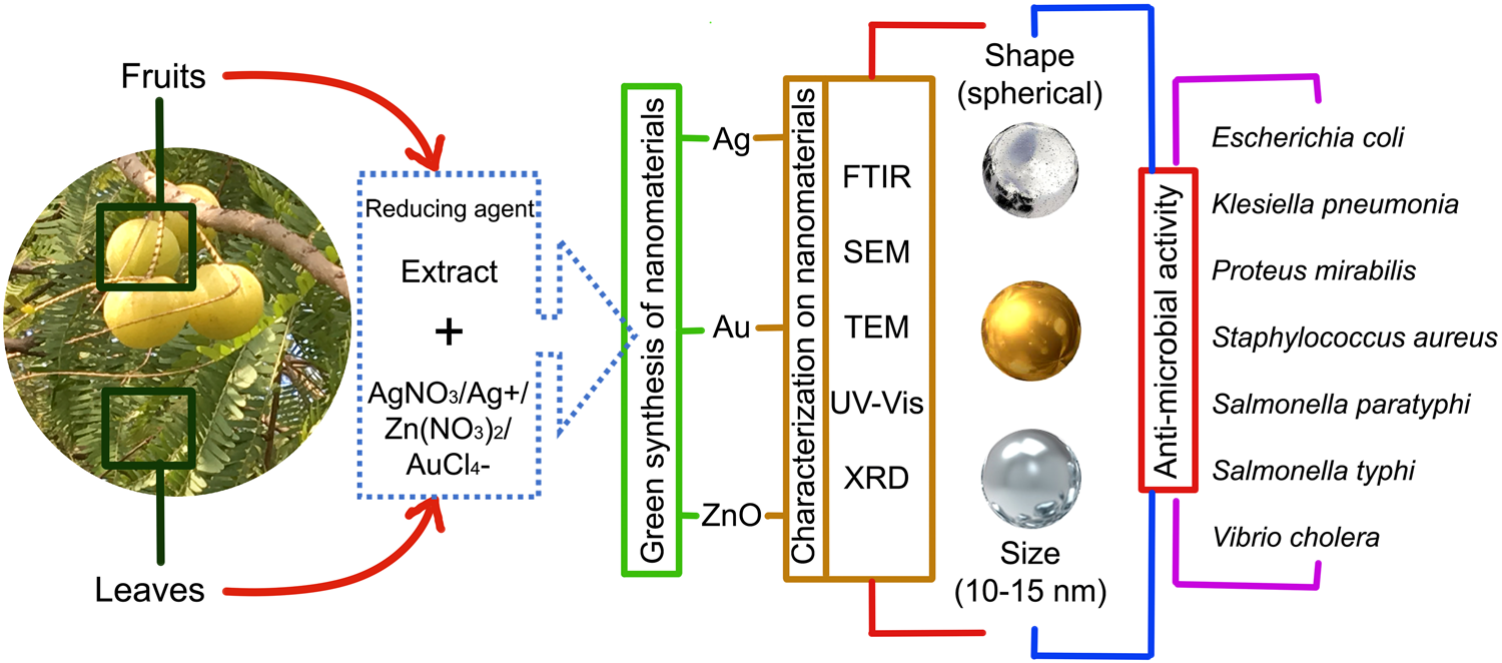
Diagram representing the green synthesis of metal/metal oxide nanoparticles from *Emblica officinalis* Gaertn. syn. *Phyllanthus emblica* L. extracts, their characterization and effects against microorganisms (*Source*: unpublished photograph of Sandeep Kumar Verma and Saikat Gantait)

In the recent years, the development of plant-based nanomaterials is significantly increased due to its simple, eco-friendly, and cost-effective approach as compared to the conventional chemical method. The green synthesized method was used for the preparation of eco-friendly silver nanoparticles (AgNPs) at various conditions (temperature, time, reducing agent, and concentrations of silver nitrate (AgNO_3_). For this, *E. officinalis* extract was used as a reducing agent (Fig. 6). Their size (41.2 nm), shape and structure were characterized by transmission electron microscopy (TEM), X-ray diffraction (XRD), scanning electron microscopy (SEM), and UV–Vis spectrophotometer (Mookriang et al. 2013). A simple, eco-friendly, low-cost, green synthesis of silver nitrate nanoparticle using *E. officinalis* fruit extract as a capping, stabilizing, and reducing agent was reported by Masum et al. (2019). Further, as-prepared AgNPs were characterized by different methods including Fourier transform spectroscopy (FTIR), TEM, X-ray diffraction (XRD), SEM, and energy-dispersive X-ray (EDX) (Fig. 6). The AgNPs were spherical in shape with particle size ranged 19.8–92.4 nm and the average diameter was 39 nm. Moreover, different concentration (5, 10, 20, and 30 μg/ml) of AgNPs used for antimicrobial (acidovorax oryzae strain RS-2 of rice bacterial brown stripe) activity and compared with control (*E. officinalis* fruit extract) group and 20 μg/ml AgNO_3_ showed remarkable antimicrobial activity (Masum et al. 2019). Maria et al. (2019) reported a facile, green synthesis method using *E. officinalis* leaf extract. The as-prepared zinc oxide nanoparticles (ZnONPs) were further characterized by XRD, FTIR, TEM, UV–Vis diffuse reflectance spectroscopy, filed emission-SEM, and photoluminescence measurements. ZnONPs are quasi-spherical with a particle size of 30–40 nm. The as-synthesized ZnONPs showed growth inhibitory effects against *Escherichia coli, Vibrio cholerae,* and *Salmonella paratyphi* (Maria et al. 2019). The green fabrication of AgNPs using fruit residue of *E. officinalis* was reported by Nayagam et al. (2018). The as-synthesized AgNPs further characterized by various techniques including UV–Vis, FTIR, XRD and SEM. The antimicrobial activities of AgNPs (spherical in shape) showed maximum growth inhibition of *Proteus mirabilis, Staphylococcus aureus, Salmonella typhi*, and *Vibrio cholera* (Fig. 6). The rapid, green route of fabrication of silver (Ag) and gold (Au) NPs using *E. officinalis* fruit extract (as a reducing agent) was demonstrated by Ankamwar et al. (2005). TEM analysis revealed that Ag and Au nanoparticles size ranged from 10 to 20 nm and 15 to 25 nm, respectively. In another study, AgNPs were synthesized from a simple, fast, eco-friendly, and green synthesis approach using *E. officinalis* fruit extract. SEM analysis result showed that AgNPs spherical shaped and particle size ranges between 19 and 45 nm with an average size of 30 nm. The as-prepared AgNPs exhibited remarkable antibacterial effects including *Staphylococcus aureus* and *Klebsiella pneumonia* bacteria (Renuka et al. 2020). These studies provide evidence about green synthesized nanomaterials can be potential candidates for antibacterial activities. Furthermore, these nanomaterials could be useful agents for biotechnological and agricultural applications.

## Conclusion

The achievements in the field of in vitro propagation-based research on *E. officinalis*, since the 1980s till 2020, have been highlighted in this review. There are merely a few reports on the direct, indirect organogenesis and somatic embryogenesis of this tree with high medicinal value; and there is still a lot of research work that still needs to be explored on the concerned topic (Fig. 5). For instance, the use of different basal media such as Eeuwen’s medium (Eeuwen 1976), Nitsch and Nitsch medium (Nitsch and Nitsch 1969), Schenk and Hildebrandt medium (Schenk and Hildebrandt 1972), White’s medium (White 1963), etc. can also be attempted as there is a report on lesser leaching of phenolic compounds and higher shoot proliferation frequency in these media. For carbon source, only sucrose was taken into account for *E. officinalis*; whereas, other derivatives like fructose, galactose, maltose, and other complex polysaccharides or sugar alcohols like mannitol can also be considered. There is no report on the use of urea derivatives like thidiazuron and topolin group (like *meta*-topolin) of growth regulators in *E. officinalis* as their use can serve as better replacements of regular cytokinins (like BA or Kn) for direct and indirect organogenesis. Synthetic derivatives like dicamba that have auxin-like activity can also be explored to attain high-frequency callus induction. The use of additives like adenine sulfate and activated charcoal for enhancing shoot and root growth, respectively, is yet to be tested on this tree. The concentration of ascorbic acid, citric acid and PVP in the basal media needs to be standardized for minimizing the leaching of phenolic compounds and browning of tissues. The enhancement of core secondary metabolite production in *E. officinalis* needs to be addressed adequately. Escalated production of emblicanin-A and emblicanin-B can be attained via cell suspension culture, added with elicitors of biotic and abiotic origin. Genetic transformation like hairy root culture via *Agrobacterium rhizogenes* serves as a promising tool for enhancing the metabolite profile of any medicinal plant (Gantait and Mukherjee 2021a) and this strategy is yet to be explored in *E. officinalis*. Other novel genetic transformation approaches like electroporation (direct) method or indirect via vector mediated using *A. tumifaciens* or other related vectors are left unexplored to date. Polyploidy can also be attempted towards enhancing secondary metabolites (Gantait and Mukherjee 2021b), which can be achieved via the use of colchicine and other anti-mitotic chemicals like oryzalin or trifluralin. In vitro mutagenesis can also serve as a viable option for the amelioration of emblicanin-A and -B biosynthesis via the use of chemical like ethyl methane sulphonate and ethidium bromide or using physical mutagens such as ultra-violet or gamma radiation. Owing to its nutritional and pharmaceutical values, nanotechnology can also be explored in the near future. Henceforth, this review brings forth all the in vitro biotechnological work done in *E. officinalis* till date and cites the significant shortcomings that may serve as a base for further advanced experimental work.

### Prospect of *E. officinalis* under cutting-edge biotechnological interventions

In addition to all the aforementioned research investigations, numerous research studies are required for the evaluation through the appropriate interventions of some biotechnological tools such as transgenic technology, marker-assisted selection, quantitative trait loci (QTL), functional genomics, RNA interference, proteomics, etc. These tools easily facilitated the route for booming utilization and amalgamation of different scientific areas, which positively necessities the deliverance of quality research for tree biotechnologies. Apart from this, system-based nano-technological instruments concentrate and deal with some important issues (Verma et al. 2018, 2019) related to regular farming of varieties of trees species. This eventually impacts and helps in the enhanced transformation of agroforestry sector. Successful and prosperous growth and incorporation of such scientific areas leads to the enormous hammer in different ways. Finally, this phenomenon generates new, fruitful, and essential data by providing necessary supports as well as contributions in electrifying projections and scenarios to create opportunities in biotechnological improvement of tree species at the global level.
